# What do temporal lobe epilepsy and progressive mild cognitive impairment have in common?

**DOI:** 10.3389/fnsys.2014.00058

**Published:** 2014-04-16

**Authors:** Yvonne Höller, Eugen Trinka

**Affiliations:** Department of Neurology, Christian Doppler Medical Centre and Centre for Cognitive Neuroscience, Paracelsus Medical UniversitySalzburg, Austria

**Keywords:** epilepsy, mild cognitive impairment, subjective cognitive complaints, memory decline, neuroimaging, neurophysiology

## Abstract

Temporal lobe epilepsy (TLE) and mild cognitive impairment (MCI) are both subject to intensive memory research. Memory problems are a core characteristic of both conditions and we wonder if there are analogies which would enrich the two distinct research communities. In this review we focus on memory decline in both conditions, that is, the most feared psychosocial effect. While it is clear that memory decline in MCI is highly likely and would lead to the more severe diagnosis of Alzheimer's disease, it is a debate if TLE is a dementing disease or not. As such, like for MCI, one can differentiate progressive from stable TLE subtypes, mainly depending on the age of onset. Neuroimaging techniques such as volumetric analysis of the hippocampus, entorhinal, and perirhinal cortex show evidence of pathological changes in TLE and are predictive for memory decline in MCI. Several studies emphasize that it is necessary to extend the region of interest—even whole-brain characteristics can be predictive for conversion from MCI to Alzheimer's disease. Electroencephalography is increasingly subject to computational neuroscience, revealing new approaches for analyzing frequency, spatial synchronization, and information content of the signals. These methods together with event-related designs that assess memory functions are highly promising for understanding the mechanisms of memory decline in both TLE and MCI populations. Finally, there is evidence that the potential of such markers for memory decline is far from being exhausted. Similar structural and neurophysiological characteristics are linked to memory decline in TLE and MCI. We raise the hope that interdisciplinary research and cross-talk between fields such as research on epilepsy and dementia, will shed further light on the dementing characteristics of the pathological basis of MCI and TLE and support the development of new memory enhancing treatment strategies.

## 1. Introduction

Memory problems are a core symptom of amnestic mild cognitive impairment (MCI) (Stewart, 2012a), but are also one of the chief complaints of patients with temporal lobe epilepsy (TLE) (Helmstädter, 2002; Butler and Zeman, 2008). Even worse, quality of life is negatively influenced by memory impairment (Fisher et al., 2000). This superficial analogy is not only a possible link between TLE and MCI but can serve as starting point from which researchers in each field may add innovative aspects to their respective research areas. Detection of early prognostic markers of memory deficits in MCI and TLE would obviously pave the way for new therapeutic programs, for memory augmentation, and treatment of memory deficits, possibly reducing the overall prevalence of memory disorders and improving quality of life in these patients (DeKosky and Marek, 2003; Geda, 2012).

Current research endeavors are focused on early diagnosis of MCI and TLE. In the case of MCI it is clear that neuropsychological tests alone are not sufficient, since they are not sensitive enough for patients with subjective complaints and no significant and clinically detectable deficits (Stewart, 2012b). Analogously, electroencephalographic assessment and imaging may lead to dubious findings, or no findings at all, in the epileptogenesis or latency period of epilepsy (Engel et al., 2013). Because of the various aetiologies and pathologic processes that may lead to memory impairment, it is suggested that a combination of several biomarkers is necessary to provide an early diagnosis with reliable prognostic validity (DeKosky and Marek, 2003). In this review, we want to highlight promising biomarkers from a pool of structural and electroencephalographical measures for which prognostic validity was evidenced for one of the two clinical groups with similar cognitive impairments, TLE and MCI. While prediction of memory decline and conversion to Alzheimer's disease is an established field of research, prognosis of memory decline in TLE mainly focuses on the prediction of post-surgical memory impairment and only rarely on the prediction of change over the course of the disorder; in this review, we focus on the latter case. As such, we identify commonalities between the two disorders, which could give rise to new approaches in research.

## 2. Neuropsychology and prognosis

TLE is most often associated with impaired long-term memory (Hermann et al., 1997). In addition to problematic long-term memory, MCI is especially annoying for patients because of impairment of prospective memory (van den Berg et al., 2012). Patients with epilepsy rank cognitive dysfunction highest among the problems experienced (Fisher et al., 2000). However, in many cases such impairments are not detected by standard neuropsychological tests of memory functions, so that complaints about memory decline commonly mismatch with test performance (Butler and Zeman, 2008). Specifically, standard test intervals ranging from some hours to days are too short to detect impaired long-term memory. Indeed, it is the episodic memory which is affected in TLE (Helmstädter, 2002). In addition, it seems difficult to tell from neuropsychological tests alone if patients are already impaired during encoding or if they just fail to retrieve learned information. Similarly, it is necessary to distinguish free recall of memories from recognition. Thus, research should take into consideration the existence of inter-individual differences with respect to the type of impairment, i.e., encoding or retrieval, recall or recognition, within the specific pathologies.

Epileptic seizures might trigger neurodegenerative changes leading eventually to memory impairments (Helmstädter, 2002; Stefan and Pauli, 2008). However, the question whether TLE is a dementing disease due to seizure activity is under debate (Helmstaedter and Elger, 1999, 2009; Jokeit and Ebner, 1999, 2002; Dodrill, 2004; Elger et al., 2004; Mantoan et al., 2009; Gonzalez et al., 2012). The typical course of epilepsy includes in the early phase a long silent period after the brain insult before recurrent seizures start, which then allows an accurate diagnosis (see Najm et al., 2001, for a review on epidemiology and risk factors). The early evolution is often caused by a structural lesion, that is, the epileptogenic lesion (Rosenow and Lüders, 2001). In fact, memory deficits correlate well with the age of the early precipitating event. This might produce an artificial correlation of memory impairment with the duration of epilepsy (Kaaden and Helmstaedter, 2009). For example, no difference between the extent of memory deficits at the time of the first diagnosis and a 5-year follow up was found (Äikiä et al., 2001), suggesting a non-progressive course of memory deficits in TLE. In fact, Helmstaedter and co-workers found that memory decline which is specific to TLE occurs in childhood and early adolescence, while a further progress of loss of memory functions runs in parallel with normal aging (Helmstaedter and Elger, 2009). On the other hand, deficits are more severe in patients with chronic TLE as compared with newly diagnosed patients (Äikiä et al., 2001). Similarly, there is a correlation between time and severity of memory impairments for patients with long chronic course (>27 years) but only for those with more than 10 secondarily generalized seizures per year, and not for complex-partial seizures (Stefan and Pauli, 2002). There are further studies suggesting that patients with a longer duration of refractory TLE exhibit more severe cognitive impairments (Hattiangady and Shetty, 2008; Stefan and Pauli, 2008; Mantoan et al., 2009). However, a longitudinal study over at least 10 years at the stage of severe, chronic and intractable epilepsy showed that duration has less influence on cognitive decline than what was found in cross-sectional studies (Thompson and Duncan, 2005). Most importantly, in this study it was demonstrated that in refractory epilepsy a high frequency of tonic-clonic seizures was the strongest predictor for cognitive decline. However, it is necessary to disentangle the influence of the underlying causes, such as mitochondrial dysfunction or inflammatory causes from seizure activity (Helmstaedter, 2007). It is obvious that progressive brain diseases, such as a malignant brain tumor, lead to a progressive pathology of memory function and it is therefore important to provide an accurate diagnosis which allows identification of progressive comorbidity.

Epileptic seizures can occur also at the stage of MCI or early Alzheimer's disease (Vossel et al., 2013) and the initial diagnosis of late-onset TLE, more specifically the epileptic amnesic syndrome, can be indicative for development of MCI and Alzheimer's disease (Cretin et al., 2012). Most interestingly, Vossel and co-workers reported that cognitive decline is detectable in patients with amnestic MCI or Alzheimer's disease on average 6.8 (MCI) or 5.5 (Alzheimer's disease) years earlier in the case of comorbid epilepsy (Vossel et al., 2013). It is important to note that epileptic symptoms predated memory symptoms or at least showed up contemporaneously.

Finally, memory deficits in TLE may be caused by clinical or subclinical seizure activity, structural, or other underlying brain pathology, adverse effects of anticonvulsant medication, and psychological mechanisms (Butler and Zeman, 2008; Hermann et al., 2010). Helmstaedter suggests that assessing two types of patients could explain contributory mechanisms of memory decline (Helmstädter, 2002). The first type includes newly diagnosed TLE patients at first presentation, i.e., after experiencing first seizures. The second type includes TLE patients with chronic course and drug resistant seizures. A follow-up measurement of memory performance should help to answer the question whether memory deficits are progressive or not. However, examining newly diagnosed TLE patients is a challenge. After the first or second seizure, a patient is usually not subjected to cost-intensive, extensive examinations, such as long-term video-monitoring which is the gold standard to reliably establish a diagnosis. Instead, in clinical practice an appropriate first line medication is chosen in order to control seizures, even if the type of epilepsy is not fully elucidated. Again, simple and reliable biomarkers to ascertain diagnosis at this early stage are highly warranted. For example, in a large multicenter study, hippocampal T2 hyperintensity and impaired hippocampal growth were found to be an early biomarker for epilepsy after febrile status epilepticus in children (Lewis et al., 2013).

A similar challenge is met by family doctors with respect to MCI. The first examination in primary care needs to reliably distinguish between age-associated memory impairment (Hänninen and Soininen, 1997), subjective memory complaints, and MCI (Stewart, 2012a,b). Classification is performed according to the global deterioration scale for aging and dementia (Reisberg et al., 1982). This scale stages individuals into Level 1, being free of both subjective and objective clinical deficits, Level 2, having subjective deficits only, Level 3, having subtle deficits in cognition and some impairments in executive functioning, affecting complex occupational and social activities, and finally Level 4, having clear deficits in cognition and functioning with reduced performance in instrumental activities of daily life (Gauthier et al., 2006). Thus, patients which present memory complaints in primary care without fulfilling the criteria for MCI are considered to suffer from so-called subjective memory complaints. These complaints are not related to significant deficits on neuropsychological scales but could still be an early form of a neurodegenerative disorder since this diagnosis implies high risk to experience further decline toward MCI (Reisberg et al., 2010). It contrast, it is possible that a patient who is diagnosed as suffering from subjective cognitive complaints has simply detected an age-associated memory impairment, which can be a symptom of normal aging. As such, subjective complaints do not necessarily correlate with progress of decline (Förstl et al., 1995). However, the diagnosis of age-associated memory impairment does not imply that the patient's memory performance is normal (Goldman and Morris, 2001). A normal test-performance can still be seen after a significant decline when the patient's starting level of memory was high. As such, neuropsychological tests cannot identify progressive memory decline alone.

If a patient has ascertained the diagnosis of MCI, the diagnosis can be further refined into amnestic, multi-domain and single, non-memory-domain subtypes (Winblad et al., 2004), as well as into a stable and a progressive group. While 30–50% of newly diagnosed MCI patients do not experience further decline of cognitive functions over 3–5 years, or even return to normal, the residual 50–70% show progress toward a more severe form of impairment (Rossini et al., 2007). This broad range of progression rates results from a large number of studies, in which inclusion and exclusion criteria varied to some degree. It is possible that correlating neuropsychological progression to specific biomarkers could yield less variable estimations of conversion rates. Therefore, the following sections of this review are dedicated to promising biomarkers in the fields of neuroimaging and neurophysiology. Finally, we close with an outlook on possible strategies for augmentation of memory function with respect to the discussed biomarkers.

## 3. Neuroimaging

Neuroimaging is of high value for diagnosis and prognosis of neurodegenerative diseases (Borghesani et al., 2010) such as MCI (Winblad et al., 2004). A characteristic symptom in MCI is atrophy of the medial temporal lobe, including memory related structures such as the hippocampus. Brain atrophy correlates with the progression of cognitive decline at the stage of subjective cognitive complaints (Förstl et al., 1995). More specifically, medial temporal lobe atrophy is able to predict progression to dementia in patients with MCI (Korf et al., 2004) with a reasonable predictive value (positive and negative predictive values 0.44 and 0.91, respectively Geroldi et al., 2006).

Decreased volumes of hippocampal formations are also a common finding in TLE patients. There is a relation between degree of memory impairment and seizure frequency, but it is suggested that structural pathologies in the temporal lobe rather than seizures cause memory difficulties (Butler and Zeman, 2008). However, these pathologies may be the consequence as well as the cause of seizures (Mathern et al., 2002a) and there are controversial findings in these respects. While neuronal cell loss in the hippocampus is not related to duration of the epilepsy, decreased neuronal density in the dentate gyrus evidenced by histology correlates positively with memory impairment in patients with TLE (Pauli et al., 2006). In addition, TLE is also associated with reduced neurogenesis in this region, further contributing to the decreased neuronal density (Mathern et al., 2002b). Neurogenesis of functional granule cells in the dentate gyrus of the hippocampus might facilitate hippocampal-dependent learning and episodic memory (Hattiangady and Shetty, 2008; Kuruba et al., 2009). On the other hand, some studies have indicated a correlation between epilepsy duration and neuronal loss (Mathern et al., 1995, 2002a). In other words, reduced neurogenesis and/or neuronal loss in this region in the chronic stage of TLE may be closely related to impairments in learning, memory, and other cognitive functions. In fact, it seems that the origin of memory problems in TLE is in childhood or adolescence (Helmstaedter and Elger, 2009). Well in line with this, hippocampal sclerosis is associated with poorer performance independent of age. However, temporal lobe atrophy is not present in all TLE patients. (Bernasconi et al., 2000) averaged six slices containing the head, body, and tail of the hippocampus. They found that the obtained hippocampal T2 relaxation times better predicted the epileptic focus than analyzing atrophy. Most interestingly, in addition to hippocampal damage, entorhinal lesions seem to play a special role in memory impairment in TLE. Especially lesions in layer III which are frequently found in TLE (Schwarcz and Witter, 2002). These findings and the importance of the rhinal cortex for memory consolidation (Axmacher et al., 2008) suggest that extracting biomarkers of the rhinal cortex may shed further light on memory problems in TLE. Thus, it is very likely that also other characteristics and other regions need to be addressed in the debate of whether memory decline in TLE is progressive or not.

Since hippocampal volumes discriminate patients with Alzheimer's disease and correlate with episodic memory performance, the volume and shape of this structure has the potential for a valid biomarker (Mueller et al., 2012). However, the hippocampus is not the only relevant structure in memory deficits. Research in MCI and Alzheimer's disease suggests that it is possibly not even the most relevant one. Volumetry of the hippocampal formation with Magnetic Resonance Imaging (MRI) revealed a relative risk of 0.69 for transition from MCI to Alzheimer's disease (Jack et al., 1999). A recent review suggests that perirhinal lesions have a stronger impact on memory functions than hippocampal lesions (Salig, 2009). Thus, atrophy of both structures, hippocampus and entorhinal cortex, may be a better marker for MCI, than either one of these alone (Winblad et al., 2004). Similarly, for TLE patients, entorhinal and perirhinal cortices are reduced in volume. Atrophy of the entorhinal cortex ipsilateral to the seizure onset zone is only found in patients with TLE, but not in other forms of epilepsy (Bernasconi et al., 2003). In addition, there is evidence that a global cortical atrophy marker such as widening of cerebrospinal fluid spaces might also be predictive for conversion from MCI to Alzheimer's disease (Teipel et al., 2007). Well in line with this, in MCI and Alzheimer's disease brain atrophy is prominent in the medial temporal lobe but also widespread over posterior cingulate and neocortical temporoparietal regions (Fox et al., 2001). Similarly, gray-matter decrease in the hippocampal area, inferior and middle temporal gyrus, posterior cingulate, and precuneus is greater in patients who convert from MCI to Alzheimer's disease than in non-converters (Chételat et al., 2005). Moreover, entorhinal and perirhinal cortices reveal reduced numbers of cells before the hippocampus is affected by degeneration (Dickerson and Sperling, 2008). The special role of the enthorinal cortex is further supported by a study which revealed that best classification accuracy of MCI was based on enthorinal cortex volume and best classification accuracy of Alzheimer's disease was based on hippocampal volume (Pennanen et al., 2004). Similarly, enthorinal cortex thickness was found to predict further memory decline in established Alzheimer's disease (Velayudhan et al., 2013). Thus, it would be worth analyzing more globally defined markers for atrophy or different combinations of regions, depending on the questions being asked.

However, by interpreting the role of hippocampal volumetry for predicting memory decline we have to consider the large variation between protocols for the delineation of the hippocampus in MR images. Up to date, there is no consistently applied standard which would allow comparing results of different studies to each other. Major differences refer to inclusion and exclusion of hippocampal white matter, definition of the anterior hippocampal—amygdala border, definition of the posterior border, and the extent to which the hippocampal tail is included, definition of the inferior medial border of the hippocampus, and use of varying arbitrary lines (Konrad et al., 2008). Therefore, it is difficult to estimate the real validity of volumetry in predicting memory decline. Alternatively, hippocampal shape features may be used instead of hippocampal volumetry. (Gerardin et al., 2009) utilized these shape features to train a support vector machine in order to perform multidimensional classification. The resulting classification rates were 94% for patients with Alzheimer's disease and 83% for MCI patients. Regarding the entorhinal cortex, reliable findings support stability of cortical thickness, volume, and surface area in normal aging (Lemaitre et al., 2012). The average cortical thickness over a region is a feature which is mostly independent from defined borders. Finally, to account for global atrophy, measures of intensity and deformation were of high predictive value for memory decline (Duchesne et al., 2010). Similarly, whole brain atrophy measures are predictive for conversion from MCI to Alzheimer's disease (Jack et al., 2005; Spulber et al., 2010).

Another promising MR technique is Diffusion Tensor Imaging (DTI). The potential of DTI for diagnosing MCI and its ability to predict further decline might have been underestimated so far, possibly due to a higher variability of fractional anisotropy than volumetric measures (Mueller et al., 2012). Indeed, DTI has been shown to be superior to hippocampal volumetry in distinguishing MCI from healthy controls (Muller et al., 2007). Hippocampal diffusivity predicts conversion from amnestic MCI to Alzheimer's disease at least as well as hippocampal atrophy (Kantarci et al., 2005; Fellgiebel et al., 2006).

## 4. Neurophysiology

Clinical EEG is the standard neurophysiological test in patients with epilepsy, with a high positive and low negative predictive value of epileptiform discharges, such as spikes and sharp waves in routine recordings. While the literature on EEG-biomarker for localization of the seizure-onset zone is overwhelming, the information about memory-relevant biomarkers is scarce, and there is even less literature about prediction of memory decline.

Analysis of peak-frequency showed that poorer memory performance coincides with lower alpha peak (Ripper et al., 2001). Similarly, in children with epilepsy the differential activation of memory-resources has been documented with event-related power changes in the theta and lower-alpha range (Krause et al., 2008). Thus, assessing frequency properties of the EEG in TLE patients may reveal memory-relevant features. As such, using the rat pilocarpine model of TLE, it was found that spatial memory declines soon after status epilepticus and that these deficits correlate with a decrease of theta power but not with interictal-like activity in the hippocampus (Chauviere et al., 2009). Similarly to results from cross-sectional studies in humans (Helmstaedter and Elger, 2009), the loss of spatial memory ability is stable and not progressive in this model.

Intracranial EEG is only used in presurgical assessment to better delineate the seizure onset zone (Foldvary-Schaefer, 2004) and cannot be applied in the early stages of TLE. Indeed, neurophysiological parameters have been underutilized to assess the functional deficit zone, including memory deficits in patients with TLE (Grunwald and Vannucci, 2004). Event related designs, assessing brain signals in response to items that have to be memorized and recalled, are well suited to identify abnormal patterns in the surface and intracranial EEG of patients. We suggest that it would be of interest if such designs could help identifying abnormal changes over time in patients with TLE.

Despite the fact that the use of EEG for diagnosis of Alzheimer's disease or MCI is not a standard in clinical practice, a large number of studies succeeded in identifying markers that distinguish patients with Alzheimer's disease from MCI. EEG-biomarkers also successfully differentiated MCI from healthy subjects (see Rossini et al., 2007, for a review). Dauwels et al. (2010) summarize characteristics of the EEG in these clinical groups. First, the EEG is dominated by slower frequencies (Bonanni et al., 2008). Fast Fourier Transformations (FFTs) show a relative increase of activity below 8 Hz and decrease above this range. This characteristic was the first measure used in quantitative EEG-evaluation in MCI patients (Prichep et al., 1994) and therefore probably the most well known in the field. However, this slowing is possibly caused by perturbations in synchronization and decreased neural complexity (Cantero et al., 2009), representing two characteristic features of EEG in MCI. Synchrony can be expressed as Pearson's correlation coefficient, coherence, Granger causality, information-theoretic and state space based synchrony measures, phase synchrony indices, stochastic event synchrony, spatial distribution of phase synchrony, and small world network characteristics, among others (see Dauwels et al., 2010, for a review). Different measures of synchronization may be increased or decreased in MCI depending on frequency range, type of analysis, and regions being assessed (Jelic et al., 2000; Stam et al., 2003; Pijnenburg et al., 2004; Koenig et al., 2005; Babiloni et al., 2006). In addition, analysis of complexity of EEG signals is a valuable approach for resting EEG (Stam, 2005). Therefore, we suggest to apply complexity analysis for prognostic assessments. For example one could use approximate entropy, auto mutual information, sample entropy, multiscale entropy, Lempel-Ziv complexity, Hjorth parameters, Petrosian fractal dimension, or Higuchi fractal dimension (see also Bao et al., 2009; Dauwels et al., 2010).

It is remarkable that assessing frequency characteristics of MCI patients differentiates progressive from stable patients. It was found that a reduction of alpha power over posterior leads is characteristic for the progressive subgroup (Luckhaus et al., 2008). Despite this success, it is acknowledged that markers of resting EEG largely overlap between progressive MCI and stable MCI (Giannakopoulos et al., 2009). For example, it was shown that measures based on frequency analysis are valuable approaches for diagnosis and they change over the course of progression, but have no prognostic validity at baseline (Jelic et al., 2000). Thus, such markers (i.e., theta and beta power) have to be recorded longitudinally to detect changes which correlate with cognitive decline. It is suggested to use event-related EEG dynamic analysis to examine neocortical circuits and neuronal networks in order to predict memory decline (Giannakopoulos et al., 2009). The major advantage of doing memory research using event related designs in the EEG is the high temporal resolution, showing immediately pathologically delayed responses. In fact, it was found that delayed components of the event-related potential differ between stable and progressive MCI (Missonier et al., 2007). As such, it is highly likely that the combination of frequency, complexity, and synchrony characteristics in event-related EEG can shed further light on the progressive nature of MCI and eventually also of memory characteristics in TLE.

Since both TLE and MCI have impaired long-term memory, it is reasonable compare the mechanisms of successful and non-successful memory formation. A large number of studies have aimed at identifying the markers in the event related potential. The studies looked at efficient encoding which was recorded during the patient's learning of items. This was compared to the patient's recall performance in a later session (Fernández et al., 2002; Voss and Paller, 2007). Another approach refers to the dual route theory of recognition, that is, familiarity and recollection components (see Rugg and Curran, 2007, for a review). Familiarity is impaired in Alzheimer's disease but to some extent preserved in amnestic MCI while recollection is impaired in both clinical groups (Ally et al., 2009). However, other research groups report that familiarity is preserved in Alzheimer's disease and MCI for pictures (Westerberg et al., 2006), and that familiarity is impaired to at least the same extent as recollection in amnestic MCI, distinguishing MCI from normal aging population but not from Alzheimer's disease (Wolk et al., 2008). These findings from functional research are well in line with decreased volume of the perirhinal cortex and the hippocampus. While the perirhinal cortex mediates familiarity, the hippocampus is considered a core region for recollection (Turriziani et al., 2008; Brown et al., 2010). Thus, when designing event-related EEG studies the dimensions of familiarity and recollection, as well as encoding and retrieval, have to be addressed.

## 5. Augmentation of memory function

There are several strategies to augment human memory, and some of these are candidates for future treatment strategies in the here discussed patient populations. Madan (2014) discusses nootropic agents, brain stimulation, mnemonic strategies, and external aids as possible approaches to support memory. These strategies include common ones such as caffeine and notes, but also implanted devices which stimulate deep brain structures electrically. The hippocampus is an obvious target for memory stimulation. For example, it was found that working memory in rats could be transferred from one animal to the other by applying hippocampal firing patterns via electrical stimulation (Deadwyler et al., 2013). Deep brain stimulation in the entorhinal cortex of epilepsy patients enhanced spatial memory (Suthana et al., 2012). Similarly, in-phase stimulation during long-term encoding in the rhinal cortex and the hippocampus of epileptic patients modulated memory performance (Fell et al., 2013) and stimulation in the fornix of Alzheimer's patients activated entorhinal and hippocampal regions, which lead to improved memory (Laxton et al., 2010). Thus, it is not surprising that Alzheimer's disease and temporal lobe epilepsy were mentioned as possible target for deep brain stimulation and memory enhancement (Suthana and Fried, 2014).

However, these stimulation techniques are still at an experimental stage and can't be used in every single patient. Specifically, most stimulation studies were performed in epilepsy patients with intracranial electrodes implanted for pre-surgical evaluation. In contrast, cognitive intervention is an established strategy for memory augmentation in MCI (Rapp et al., 2002; Simon et al., 2012). The changes are not only measurable by the assessment of memory function, but also with fMRI (Simon et al., 2012), and can be enhanced by incorporating emotional content into the training (Broster et al., 2012). While there have been several endeavors to support the validity of cognitive training in MCI or dementia, there is still room for research in temporal lobe epilepsy.

## 6. Conclusions

Even if the debate of whether TLE is a dementing disease or if there are just a few subtypes with progressive course is still ongoing, there is a lot of literature about structural markers for impaired memory whereas the literature about EEG-markers is scarce. Valid biomarkers, which can reliably predict conversion from MCI to Alzheimer's disease, could shed new light on the question of whether TLE is a disorder with progressive memory decline. It is likely that memory decline occurs in TLE patients with early onset but after a certain age does not result in a progressive course. There may be elderly patients with MCI-like symptoms who instead suffer from late-onset epilepsy and could be misdiagnosed. The biomarkers summarized in this review could help determine the mechanisms of memory loss at early-onset of TLE and, most importantly, identify patients with late-onset TLE.

Prediction of conversion from MCI to Alzheimer's disease has been paid much attention so far, but TLE research and specifically intracranial recordings in presurgical evaluations may help to find new biomarkers for both disorders. For example, the importance of the rhinal cortex for memory consolidation has been shown by use of intracranial EEG in TLE research and can be translated into prediction of memory loss in MCI by use of structural imaging. As such, functional EEG is a promising approach which should be paid more attention in the future, since it allows one to combine the strength of neuropsychological assessments and the physiological assessment of brain function at a high temporal resolution.

Finally, the mentioned structures could be target for stimulating interventions such as deep brain stimulation in the rhinal, entorhinal, and hippocampal regions. The basis for these stimulation studies are the knowledge about the functional relevance of the mentioned structures but also about the involved EEG-oscillations. While deep brain stimulation is at an experimental stage we should not forget about the good effects of cognitive intervention, being established in MCI and having the potential of enhancing memory function in TLE patients.

## Author contributions

Conception, decision on which references to include, revising, and approving of this work was carried out by all authors. The first author wrote the manuscript in accordance with the intellectual input of all authors. All authors agree to be accountable for all aspects of the work in ensuring that questions related to the accuracy or integrity of any part of the work are appropriately investigated and resolved.

## Funding

The presented research was funded by the Austrian Science Fund (FWF): KLIF 12-B00.

### Conflict of interest statement

The authors declare that the research was conducted in the absence of any commercial or financial relationships that could be construed as a potential conflict of interest.
